# Immunization of Knock-Out α/β Interferon Receptor Mice against High Lethal Dose of Crimean-Congo Hemorrhagic Fever Virus with a Cell Culture Based Vaccine

**DOI:** 10.1371/journal.pntd.0003579

**Published:** 2015-03-11

**Authors:** Nurettin Canakoglu, Engin Berber, Sukru Tonbak, Mustafa Ertek, Ibrahim Sozdutmaz, Munir Aktas, Ahmet Kalkan, Aykut Ozdarendeli

**Affiliations:** 1 Department of Microbiology, Medical Faculty, Erciyes University, Kayseri, Turkey; 2 Department of Virology, College of Veterinary Medicine, Firat University, Elazig, Turkey; 3 Department of Virology, College of Veterinary Medicine, Erciyes University, Kayseri, Turkey; 4 Refik Saydam National Public Health Agency, Ankara, Turkey; 5 Department of Parasitology, College of Veterinary Medicine, Firat University, Elazig, Turkey; 6 Department of Infectious Diseases and Clinical Microbiology, Medical Faculty, Karadeniz Technical University, Trabzon, Turkey; Florida Gulf Coast University, UNITED STATES

## Abstract

Crimean-Congo hemorrhagic fever (CCHF) is an acute tick-borne zoonotic disease. The disease has been reported in many countries of Africa, Asia, the Middle East, and in Eurasia. During the past decade, new foci of CCHF have emerged in the Balkan Peninsula, southwest Russia, the Middle East, western China, India, Africa, and Turkey. CCHF virus produces severe hemorrhagic manifestations in humans with fatality rates up to 30%. Vaccine development efforts have been significantly hampered by a lack of animal models and therefore, no protective vaccine has been achieved. Lately, IFN α/β receptor deficient (IFNAR^−/−^) mice have been established as a novel small animal model of CCHF virus infection. In the present study, we found that IFNAR^−/−^ mice highly susceptible to CCHF virus Turkey-Kelkit06 strain. Immunization with the cell culture based vaccine elicited a significant level of protection against high dose challenge (1,000 PPFU) with a homologous CCHF virus in IFNAR^−/−^ mice.

## Author Summary

The CCHF virus is one of the most geographically widespread tick-borne viruses, and has been reported in many countries of Africa, Asia, the Middle East, and in Eurasia. Since 2002, there have been more than 8000 cases in Turkey, with mortality rate around 5%, making CCHF a public health concern. There are currently no specific antiviral therapies or licensed vaccines for CCHF. Due to limitations in treatment options and difficulties posed by vector control vaccination remains the most logical method of disease control. In the present study, we showed that immunization with the cell culture based vaccine against CCHF elicited a significant level of protection against a high dose challenge (1,000 PPFU) with a homologous CCHF virus Turkey-Kelkit06 strain in IFNAR^−/−^ mice. The animals vaccinated with 5, 20, 40 μg dose of the cell culture based vaccine were partially protected (60%, 80% and 80% protection, respectively) with a significant delay in time to death. Neutralizing antibody responses are essential for the increased of protection in the mice vaccinated with the cell culture based vaccine but this cannot be the only mechanism of protection.

## Introduction

Causative agent is Crimean Congo hemorrhagic fever (CCHF) virus which belongs to the *Nairovirus* genus in the family Bunyaviridae which are enveloped viruses containing tripartite, negative polarity, single-stranded RNA [1–3]. CCHF is the most widely distributed tick-borne disease. To date, CCHF has been reported in more than 30 countries in Africa, Asia, Eastern Europe and the Middle East. A significant increase of cases in countries such as Turkey, Russia, Kosovo, Albania and Iran has recently been observed [1–4]. Geographical distribution is closely related to the presence of the primary vectors, ticks of the genus *Hyalomma* [1–6].

CCHF virus, like other tick-borne zoonotic agents, circulates in nature in tick-vertebrate-tick. The virus is transmitted to livestock and humans by the bite of infected ticks or by exposure to the tissues or blood of infected animals [1–5]. The disease is asymptomatic in infected animals with the exception of suckling mice but can develop into severe illness in humans, which are characterised by petechiae, hematemesis, extensive ecchymoses, bleeding, and hepatic dysfunction, with fatality rates up to 30%. Treatment options are limited, and supportive therapy seems to be only approach for managing the patients [1–4,7].

Currently, there is no licensed vaccine for CCHF. The only available vaccine is that produced in Bulgaria, which is made on suckling mouse brain (inactivated by chloroform, heated at 58°C, and adsorbed on aluminum hydroxide) but few data have been published and the vaccine is unlicensed by the European Medicines Agency and US Food and Drug Administration. There have been few attempts to develop a vaccine because of the sporadic and limited numbers of cases and the absence of a suitable animal model to evaluate the efficacy of vaccine candidates. The CCHF virus infects newborn mice, but an adult small animal model is necessary for studies of acquired immune responses and vaccination against the virus. IFN α/β receptor deficient mice showed marked increased sensitivity to many viruses despite the presence of an otherwise intact immune system [8,9]. Recently, IFNAR^−/−^ and STAT-1 mice have been established as adult small animal models of CCHF virus infection and were shown to be highly susceptible to the prototype CCHF virus of IbAr2000 strain [10–12]. These animal models could facilitate the study of the immune response and the testing of new vaccines against CCHF virus.

In the present study, taking advantage of IFNAR^−/−^ deficient mice as a suitable animal model, we have evaluated the protective immune responses obtained after the cell culture based vaccination and a challenge with the high dose of CCHF virus Turkey-Kelkit06 strain.

## Materials and Methods

### Ethics statement

All animal experiments were performed as specified in the regulation 5199 which describes animal protection and working with laboratory of animals in Turkey. This study was approved by Firat University ethics committee (HDEE/FU protocol 40/07), the Turkish Environmental Agency (TEA/Protocol 5199–3) and the Firat University Committee for Animal Research (CAR/FU protocol IP-1–13).

### Animals

Female 129 Sv/Ew and IFNAR^−/−^ mice, aged 7–10 weeks, were obtained from B&K Universal Ltd. (UK). Animals were maintained in an Isocage system (Allentown Inc. USA) and fed standard rodent feed and water ad libitum. All animal experiments were performed in an animal biosafety level 3 enhanced facility.

### Cells, virus and antibodies

Vero E6 (ATCC CRL-1586) cells were maintained in Dulbecco’s modified Eagle’s medium (DMEM) supplemented with 10% heat-inactivated foetal bovine serum (FBS), 100 mM l-glutamine, 50 U/ml penicillin, 50 μg/ml streptomycin (Sigma–Aldrich, Germany). The Crimean-Congo haemorrhagic fever virus Turkey-Kelkit06 strain was isolated in 2006 from the blood of a patient from the Kelkit Valley region of Turkey [13]. Virus stocks were generated by infection of confluent Vero E6 cells using a multiplicity of infection (MOI) of 0.01. At 48 h post infection (pi), or when total cytopathic effect (CPE) was visible, the cells and supernatants were harvested and centrifuged. The virus was released from the cells by three freeze and thaw cycles. Titres of the virus stocks were determined by a pseudo-plaque assay (PPA) as described previously [14]. Polyclonal rabbit and mouse anti-CCHF virus sera were generated in rabbits and mice as described previously [15]. All experiments with the live virus Turkey-Kelkit06 strain were performed in a biosafety level 3 enhanced facility.

### Assessing the infectivity of IFNAR^−/−^ mice by CCHF Turkey-Kelkit06 strain

Groups of 6 IFNAR^−/−^ mice were inoculated with containing 10,000, 1,000, 100 and 10 Pseudo Plaque Forming Unit (PPFU) of the virus by the intraperitoneal route. A group of four 129 Sv/Ew mice received 100 PPFU by the intraperitoneal route. All animals were euthanized at 2 day post infection (dpi) by isoflurane. Blood was collected by cardiac puncture in EDTA. Mice were necropsied, liver and spleen samples were collected and frozen at −80°C for virus titration. The results were recorded as the geometric mean titer (GMT) ± the standard error (SE).

### Median lethal dose

For 50% lethal dose (LD_50_) experiment, IFNAR^−/−^ mice were infected with the virus inoculates containing 10,000, 1,000, 100, 10, 5 and 2.5 PPFU by intraperitoneal route (n = 6/group). The mice were monitored twice daily for clinical score, body temperature, weight change and survival. Animals were scored for the each parameter as follows: normal (0), ruffling (1), ruffling and slight lethargy (2), lethargy (3), labored breathing and immobility (4). Mice showing clinical score 4 were considered to have reached the experimental end point and were euthanized. LD_50_ was calculated by logistic regression.

### Vaccine preparation

Confluent Vero E6 cell monolayers in T175 flasks were infected with a 0.01 MOI of CCHF virus Turkey-Kelkit06 strain and incubated for 1 h at 37°C with rocking at 10 min intervals. The inoculum was discarded, and cells were washed twice with phosphate buffer solution (PBS) and fed with DMEM containing 2% FBS. Harvest of the culture media was made day 7 post-inoculation. The cultures were freeze-thawed twice, the cell lysate was centrifuged at 4,000 rpm for 20 min, and the supernatant was collected. The bulk culture of the virus was precipitated with 0.5 M NaCl, and 10% (w/v) PEG8000 was added and the solution stirred overnight at 4°C. The mixture was centrifuged at 12 000 rpm for 30 min at 4°C. The supernatant was discarded, and the virus precipitate was dissolved in TNE buffer (50 mM Tris-HCl, 150 mM NaCl, and 5 mM EDTA, pH 7.2).

The virus-containing solution was loaded on a 20–60% sucrose gradient and centrifuged in a Beckman SW28 rotor at 25, 000 rpm at 4°C. Five hundred microliters of fractions were collected from the bottom of the tube by the fraction collector. The fractions were analyzed for the CCHF virus proteins by western blotting. The virus pool was diluted four-fold with PBS and subjected to ultracentrifugation at 24, 000 rpm at 4°C. Each virus pellet was diluted in 1 ml PBS. To prepare the inactivated cell culture based CCHF vaccine, formalin (37% formaldehyde, Sigma, USA) was diluted 1:40 and was added dropwise to the purified virus pool to give a final formaldehyde dilution of 1/2,000. The inactivation procedure was carried out for 168 h at 22°C. The absence of infectious virus was confirmed by PPA for a period of up to 7 days. The residual formalin in the samples was neutralized by the addition of one part 3.75% (w/v) sodium metabisulfite to 100 parts of the virus suspension. The neutralized virus solution was dialyzed at 4°C against PBS. After the neutralization step, the inactivated virus suspension was filtered through a 0.22μm low protein binding filter unit (Millipore, USA). The bulk vaccine was mixed with adjuvant (Imject Alum, Pierce, USA) at a concentration of 250 μg of alum per 500 μl of vaccine dose.

### SDS-PAGE and immunoblotting

The unpurified and purified vaccine antigen samples were separated on 10% resolving and 5% stacking SDS-PAGE gels in a mini electrophoresis unit (Bio-Rad, USA) at 90 V for 2 h. The protein gel was directly stained with 0.05% Coomassie brilliant blue (Sigma, USA). The purified vaccine antigen samples were transferred onto a PVDF membrane (Millipore, USA) under wet conditions using a trans-blot apparatus (Bio-Rad, USA). The membrane was blocked with 5% skimmed milk in PBS and washed three times with wash buffer (0.1% skimmed milk in PBS with 0.05 Tween 20). The membrane was incubated with a primary antibody hyperimmune serum grown in rabbit against the CCHF virus at a dilution of 1:2000 for 1 h at 37°C, followed by goat anti-rabbit alkaline phosphatase (AP) conjugated antibody (1:2000 dilution, Southern Biotech, USA). The NP and Gc-specific mouse monoclonal antibodies against IbAr10200 strain of CCHF virus were kindly provided by Dr. Schmaljohn (United States Army Medical Research Institute, Fort Detrick, Maryland USA). The Gc-specific mouse monoclonal antibody (MAb 11E7) has been shown to recognize linear epitope of Gc of IbAr10200 ([16]. Thus, the purified vaccine antigen samples were probed with the NP and Gc-specific mouse monoclonal antibodies followed by goat anti-mouse AP conjugated antibody (1:1500 dilution, Southern Biotech, USA). ECL substrate solution (Pierce ECL Western Blotting Substrate, Thermo Scientific, USA) was added to the membrane, exposed to an autoradiograph film (KODAK X-OMAT, Sigma Germany), and developed using a Kodak developer (X-OMAT 1000A, Sigma Germany).

### Immunization of IFNAR^−/−^ mice and CCHF virus Turkey-Kelkit06 strain challenge

IFNAR^−/−^ mice were divided in four groups for the experiment. Three groups (n = 10 each) were immunized via the intraperitoneal route with 5, 20, or 40 μg of the cell culture based vaccine. The control group (n = 6) was mock immunized with PBS. Booster injections with the same formulation were given on days 21 and 42 after the first immunization. Then, all IFNAR^−/−^ mice groups were challenged with 1,000 PPFU of the virus two weeks after the final immunization. The mice were monitored twice daily for clinical score, body temperature, weight change, geometric mean time to death (GMD), and survival.

### Detection of the viremia levels of the vaccinated animals

Three groups (n = 3 each) were immunized via the intraperitoneal route with 5, 20, or 40 μg of the cell culture based vaccine. The control group (n = 3) were mock immunized with PBS. Mice were immunized with three times at three weeks intervals and challenged with 1,000 PPFU of the virus two weeks after the final immunization. At 48 h post- challenge, all animals were killed humanely and samples of blood, liver and spleen were collected. Titres of the virus were determined by PPA. The results were recorded as the geometric mean titer (GMT) ± the standard error (SE).

### Titration of neutralization antibody

The neutralization titer of the sera collected from the animals was determined using a pseudo-plaque reduction neutralization assay (PPRNT) as described with minor modifications [15]. Briefly, Vero E6 cells were grown to confluence with DMEM containing 10% FBS in 24-well microtiter plates (Corning, USA) at 37°C, 5% CO_2_ for 18–24 h. The sera from the immunized and control mice were heat-inactivated and serial 2-fold dilutions were prepared and mixed with an equal volume of the Turkey-Kelkit06 virus-containing 50 PPFU The plates were incubated overnight at 4°C. Two hundred microliters of the serum/virus mixtures were adsorbed to confluent cell monolayers (in triplicate) and incubated for an additional hour at 37°C. The supernatant was removed, and the cell monolayer was overlaid with the virus medium (DMEM with L-glutamine containing 2% FBS, 2% HEPES (1 M), 1% penicillin, and streptomycin 100×) supplemented with 1% carboxymethyl cellulose (Sigma-Aldrich, Germany) and incubated at 37°C, 5% CO_2_ for 4 days. The cells were fixed with formaldehyde (Sigma-Aldrich, Germany), permeated with 0.1% Triton X 100 in PBS for 20 min, and blocked with 5% skimmed milk in PBS. The polyclonal rabbit anti-CCHF virus serum (1:1500) was added to each well in TBST (100 mM Tris-HCl, 1.5 M NaCl, 1% Tween 20) and incubated for 1 h at room temperature. After three washes for 10 min in TBST, a goat anti-rabbit β-gal conjugate diluted 1:1500 in TBST (Southern Biotech, USA) was added to each well, and the plates were incubated for 1 h at room temperature. The cells were washed five times with TBST, and the substrates nitro blue tetrazolium (Sigma-Aldrich, Germany) and X-gal (5-bromo-4-choloro-3-indolyl- beta-D-galactopyranoside, Sigma-Aldrich, Germany) were added to each well and incubated at 37°C. The microplates were microscopically examined every 10 min. The neutralizing anti-CCHF virus antibody titers were directly assigned to the highest dilution with >50% reduction. The results were recorded as the geometric mean titer (GMT) ± the standard error (SE).

### ELISA

An enzyme-linked immunosorbent assay (ELISA) was performed for the determination of specific antibodies against the CCHF virus. The viral antigens prepared from Vero E6 cells infected with the Turkey-Kelkit06 strain of CCHF virus were purified by sucrose gradient ultracentrifugation. Flat-bottomed 96-well plates (Nunc, Denmark) were coated with 100 ng/well of the viral antigen in 0.05 M carbonate bicarbonate buffer (pH 9.6) at 4°C overnight. The plate was blocked with 5% skimmed milk in PBS for 1 h at 37°C. After washing with PBS containing 0.05% Tween 20 (PBS-T), the plate was incubated at 37°C for 1 h with sera from the immunized mice serially diluted in PBS containing 1% skimmed milk. After washing with PBS-T, the plate was incubated at 37°C for 1 h with a horseradish peroxidase-conjugated polyclonal goat anti-mouse IgG (Southern Biotech, USA) diluted 1:5000. The plate was washed with PBS-T and colorized with the peroxidase substrate solution by adding 0.5 ml of 0.2 M citrate buffer, pH 3.3, and 0.25 ml of 2 mg/mL TMB to 9.25 mL of water and 25 μL of 3% hydrogen peroxide. The reaction was stopped by 1N HCl. The absorbance was read at a wavelength of 450 nm (OD450) by a spectrophotometer (Biotek ELx80, Germany). The end point of the antibody titer was determined as absorbance greater than the mean + 2 standard deviations of the negative control sera. The results were recorded as the geometric mean titer (GMT) ± the standard error (SE).

### Statistics

Scoring index, body weight, and body temperature were compared between groups to 2-way ANOVA with Bonferroni`s post-tests. The log rank (Kaplan-Meier) test was used for survival analysis and extension in time to terminal disease following CCHF virus challenge. Differences in ELISA and virus neutralization titers between groups were analyzed by 2-way ANOVA followed by Bonferroni`s post-tests. Viral levels between groups were determined by one-tailed Mann-Whitney test. All statistical analyses were performed on GraphPad Prism v5.00 (GraphPad Software, La Jolla, CA, USA).

## Results

### Susceptibility of IFNAR^−/−^ mice to CCHF virus Turkey-Kelkit06 strain

**Fig 1:**
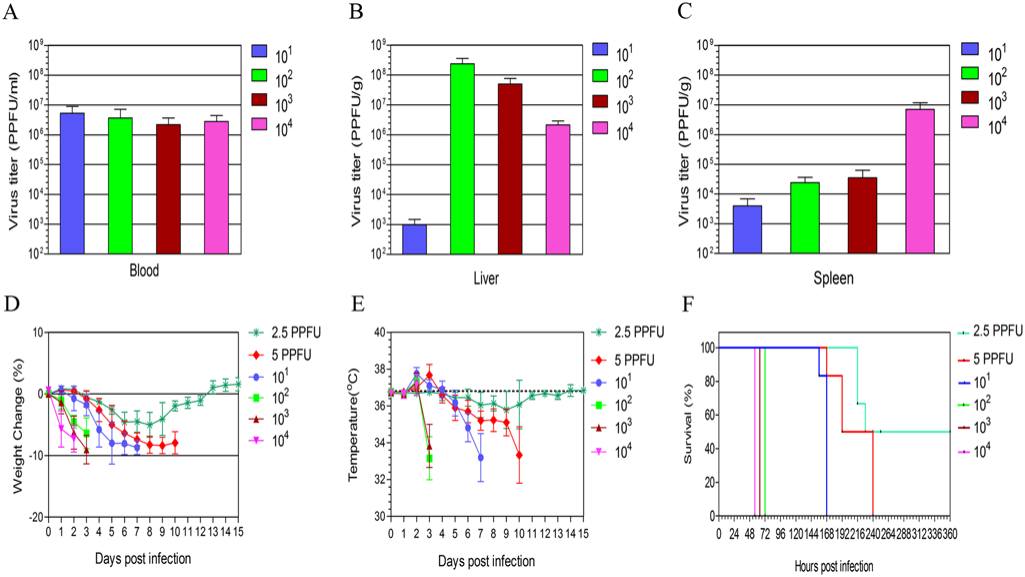
Titres of the CCHF virus Turkey-Kelkit06 strain, weight loss curves, body temperatures changes and survival of IFNAR^−/−^mice. Titres of the CCHF virus Turkey-Kelkit06 strain recovered in the blood (A), liver (B) and spleen (C) after intraperitoneal infection with 10^1^, 10^2^, 10^3^ or 10^4^ PPFUs of CCHF virus Turkey-Kelkit06 strain. All animals were sacrificed at 2 days post-infection (dpi). The virus was extracted from the blood and indicated organs, and the virus titres were determined by pseudo plaque assay (PPA). Each point represents the mean values of the viral titre of six animals, and the standard deviations are shown as error bars. The IFNAR^−/−^mice (n = 6 each) were infected with 2.5, 5, 10^1^, 10^2^, 10^3^ or 10^4^ PPFUs of CCHF virus Turkey-Kelkit06 strain. The mice were monitored twice daily for mean weight change (D), body temperature (E), and survival (F). The median lethal dose was calculated by logistical regression. The standard deviations are shown as error bars.

To determine whether IFNAR^−/−^ mice were susceptible to CCHF virus Turkey- Kelkit06 strain, IFNAR^−/−^ mice were inoculated intraperitoneally with ten-fold serial dilutions (10,000, 1,000, 100 and 10 PPFU) of the virus. All animals were euthanized at 2 dpi. The mean titres of the virus in blood, liver and spleen indicated that the virus titres are independent of the dose of the virus used for infection (Fig. 1A, 1B and 1C). We were not able to detect infectious virus in 129 Sv/Ew mice after infection with 100 PPFU of the virus. These data suggest that IFNAR^−/−^ mice are highly susceptible to CCHF virus Turkey-Kelkit06 strain.

### Median lethal dose of CCHF Turkey-Kelkit06 strain in IFNAR−/− mice

To determine the LD50, IFNAR^−/−^ mice were challenged with serial dilutions (10,000, 1,000, 100, 10, 5 and 2.5 PPFU) of the virus. Animals that received 10,000, 1,000 and 100 PPFU showed ruffling, slight lethargy, weight loss and slightly increased body temperature at 2 dpi (Fig. 1D and 1E). The general conditions deteriorated very rapidly. When reaching the experimental end point, the groups receiving 10,000, 1,000 and 100 PPFU were euthanized at 56, 64 and 72 hours pi, respectively (Fig. 1F). Animals from the 10 PPFU dose group became hypothermic at 4 dpi (Fig. 1E) and reached the experimental end point at 7 dpi (Fig. 1F). Animals that received 5 PPFU showed the signs of the disease at 6 dpi. One animal was found dead in the cage at 7 dpi, and another animal was euthanized at 192 hours pi. The remaining mice were euthanized at 10 dpi (Fig 1F). The IFNAR^−/−^ mice that received 2.5 PPFU exhibited slight lethargy and ruffling at 7 dpi. Three of 6 animals became hypothermic at 9 dpi and reached the experimental end point at 11 dpi. The remaining mice survived (Fig. 1E and 1F). We determined that the LD50 of the CCHF virus Turkey-Kelkit06 strain is as low 2.5 PPFU in IFNAR^−/−^ mice. Our data indicate that the CCHF virus Turkey-Kelkit06 strain is lethal to IFNAR^−/−^ mice and that the geometric mean time of death is dose-dependent.

### Antigenicity of the cell culture based vaccine

**Fig 2:**
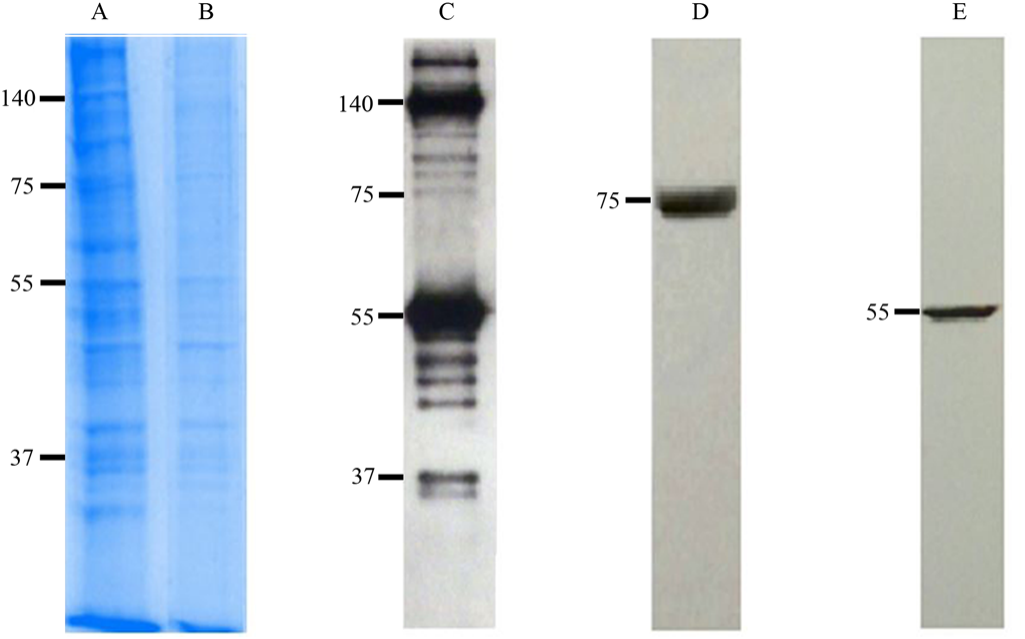
Confirmation of the antigenicity of the cell culture based vaccine by SDS-PAGE and western blotting. The unpurified vaccine antigens assessed before sucrose gradient ultracentrifugation and the purified vaccine antigens obtained after the purification and inactivation step were subjected to SDS-PAGE analysis shown in Fig. 2A and 2B, respectively. The purified vaccine samples were immunoblotted with 1:2000 dilution of the hyperimmune serum grown in rabbit against the CCHF virus and followed by goat anti-rabbit AP conjugated antibody to visualize the antigenic bands. (Fig. 2C). The cell culture based vaccine antigens were probed with anti-Gc (Fig. 2D) or anti-NP (Fig. 2E) monoclonal antibodies of the CCHF IbAr10200 virus.

The antigenicity of the cell culture based vaccine was determined by SDS-PAGE and blotting. The unpurified (Fig. 2A) and purified (Fig. 2B) vaccine antigens were electrophoresed on SDS-PAGE gel, and the purified vaccine antigens were immunoblotted with a CCHF virus Turkey-Kelkit06 virus polyclonal antibody to visualize the antigenic bands. The viral proteins PreGn (140 kDa), NP (55 kDa), Gn (37 kDa), and Gc (75 kDa) were present in the cell culture based vaccine (Fig. 2C). To determine if the cell culture based vaccine antigens obtained from the CCHF virus Turkey-Kelkit06 strain reacted with anti-Gc or anti-NP monoclonal antibodies of the CCHF IbAr10200 virus, the cell culture based vaccine antigens were probed with anti-Gc or anti-NP monoclonal antibodies of the CCHF IbAr10200 virus. Western blot analysis demonstrated two protein bands with apparent molecular masses of 75 kDa (Gc) and 55 kDa (NP) respectively (Fig. 2D and Fig. 2E), indicating that the cell culture based vaccine obtained from the CCHF virus Turkey-Kelkit06 strain may induce cross-reactive antibodies to other strains of CCHF virus.

### Immunogenicity of the cell culture based vaccine against CCHF in IFNAR^−/−^ mice

**Fig 3:**
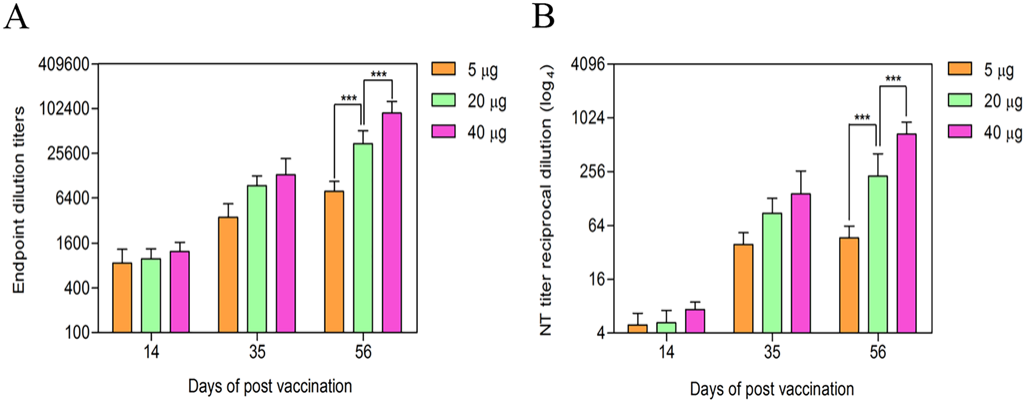
Humoral immune responses to the cell culture based vaccine against CCHF in IFNAR^−/−^ mice. Groups of 6 IFNAR^−/−^ mice were immunized three times at three weeks intervals with 5, 20, or 40 μg of the cell culture based vaccine. The sera obtain from the mice at 14, 35, and 56 days were assayed for anti-CCHFV antibody end point titre (A) and neutralizing antibody titres determined by a 50% pseudo plaque reduction neutralization assay (PPRNT) (B). The error bars indicate the standard deviation. Differences in the ELISA titres and virus neutralization titres between groups were analyzed by 2-way ANOVA followed by Bonferroni’s post test, which is denoted by asterisks. *** (p<0.001).

Three groups of IFNAR^−/−^ mice (n = 10 per group) received intraperitoneal injections of 5, 20, or 40 μg of the cell culture based vaccine. Booster doses were administered at 3 and 6 weeks after the initial immunization. Six mock-treated IFNAR^−/−^ mice were immunized with PBS on the same schedule. The first immunization induced the CCHF virus-specific IgG antibody response, and the levels of virus-specific antibodies significantly increased following booster immunizations in all groups (Fig. 3A and Table 1). The animals did not generate detectable neutralization antibodies after the first immunization (Fig. 3B and Table 1). After the second immunization, the 5, 20, and 40 μg dose groups showed neutralizing antibody titres of 1:38,4, 1:83,2 and 1:89,6, respectively (Fig. 3B and Table 1). The third immunization resulted in significantly increased levels of neutralizing antibody titres in the 20 and 40 μg dose groups 1:204,8 and 1:614,4, respectively, whereas the 5 μg dose group remained at a low level, 1:44,8 (Fig. 3B and Table 1). These results suggest that the neutralizing antibody titre induced by the cell culture based vaccine increased in a dose-dependent manner.

**Table 1:** Antibody responses, morbidity and mortality in IFNAR^−/−^ mice intraperiteonally immunized three times at three weeks intervals with 5, 20, or 40 μg of the cell culture based vaccine.

Vaccine	Dose (μg)	Antibody titer (GMT)						Morbidity^a^	Mortality^b^
		First Immunization		Second Immunization		Third Immunization			
		*ELISA	**N ab	*ELISA	**N ab	*ELISA	**N ab		
**Inactivated cell culture based**	40	1333.3±191 N = 10	***ND	13200±2533 N = 10	89.6±15 N = 10	88450± 11340 N = 10	614.4±68 N = 10	3/10 (7)	2/10
	20	1200.0±168 N = 10	***ND	9600±954 N = 10	83.2±12 N = 10	35200± 4950 N = 10	204.8±56 N = 10	5/10 (7)	2/10
	5	880.5±136 N = 10	***ND	3600±519 N = 10	38.4±4 N = 10	8000±826 N = 10	44.8±5 N = 10	8/10 (8)	4/10

The sera obtain from the mice at 14, 35, and 56 days. All animals were challenged with 1,000 PPFU (400 LD50) of the CCHF virus Turkey-Kelkit06 strain two weeks after the last immunization.

^a^ Sick mice/total mice. Numbers in parenthesis indicate cumulative days showing clinical display.

^b^ Dead mice/total mice.

N = Each group has 10 IFNAR^−/−^ mice

*Mean ± SD ELISA OD450 serum titers at 1/100 dilution.

**Mean ± SD log4 titers based on a 50% pseudo plaque reduction.

***ND: Not determined.

### Challenge studies

**Fig 4:**
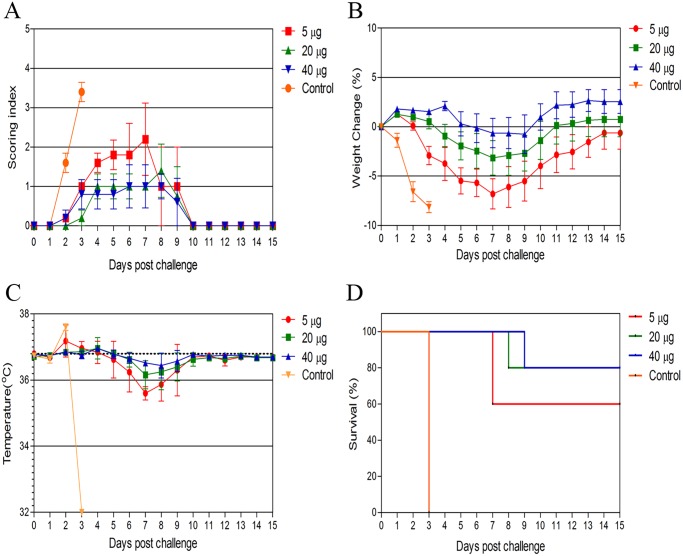
Protection of IFNAR^−/−^ mice immunized with the cell culture based vaccine against CCHF virus Turkey-Kelkit06 strain challenge. Groups of 6 IFNAR^−/−^ mice were immunized three times at three weeks intervals with 5, 20, or 40 μg of the cell culture based vaccine. The control group of IFNAR^−/−^ mice (n = 6) was mock immunized with phosphate buffered solution (PBS). All animals were challenged with 1,000 PPFU (400 LD50) of CCHF virus Turkey- Kelkit06 strain two weeks after the last immunization. The mice were monitored twice daily for the cumulative mean symptom scores (A), the daily variations in weight as percentages compared to before the virus challenge (B), body temperature (C), and geometric mean time to death and survival (D). The animals were monitored for three weeks after the challenge. The standard deviations are shown as error bars.

Following the three inoculations with the cell culture based vaccine containing 5, 20, and 40 μg, the IFNAR^−/−^ mice were challenged with 1,000 PPFU (400 LD50) of CCHF virus Turkey-Kelkit06 strain two weeks after the last immunization. Survival was assessed over a period of three weeks. The control group of 6 IFNAR^−/−^ mice showed signs of the disease, manifested as rapid weight loss at 2 days post-challenge (Fig. 4A and 4B). All became hypothermic and were euthanized at 3 days post challenge (Fig. 4C and 4D).

The IFNAR^−/−^ mice immunized with the cell culture based vaccine showed varying degrees of protection. Eight of the ten IFNAR^−/−^ mice immunized with the 5 μg dose group showed ruffling, lethargy and weight loss at 3 days post-challenge (Fig. 4A and 4B). Four of the sick animals become hypothermic at 6 days post-challenge and reached the experimental end point at 7 days post-challenge and were euthanized (Fig. 4C and 4D). The other four mice began gaining weight at 8 days post-challenge and returned to their starting weight by day 14 after the challenge (Fig. 4B). Five of the ten the IFNAR^−/−^ mice immunized with 20 μg of the vaccine exhibited slight lethargy, reduced activity and weight loss at 4 days post-challenge (Fig. 4A and 4B). Interestingly, these mice remained stable until 7 days post-challenge, but two suddenly became hypothermic (Fig. 4C) and declined quickly. Therefore, these two animals were euthanized at 8 days post-challenge (Fig. 4D). For the IFNAR^−/−^ mice immunized with 40 μg of the vaccine, only three animals exhibited ruffling and reduced activity at 7 days post-challenge (Fig. 4A). Two animals became hypothermic and were euthanized at 9 days post challenge (Fig. 4C and 4D). The animals vaccinated with 20 and 40 μg of the vaccine displayed 80% protection for the IFNAR^−/−^ mice (2/10), whereas the 5 μg dose group showed 60% survival (4/10) (Fig. 4D and Table 1). These results indicate that immunization with the cell culture based vaccine elicited a significant level of protection.

### Virus titres of vaccinated animals

**Fig 5:**
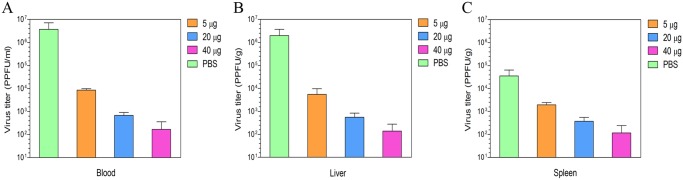
Determination of virus titres of the vaccinated animals. Groups of 3 IFNAR^−/−^ mice were immunized three times at three weeks intervals with 5, 20, or 40 μg of the cell culture based vaccine. The control group of IFNAR^−/−^ mice (n = 3) was mock immunized with phosphate buffered solution (PBS). All animals were challenged with 1,000 PPFU (400 LD50) of CCHF virus Turkey-Kelkit06 strain two weeks after the last immunization. All animals were sacrificed at 2 days post-infection. Virus was isolated from the blood (A), liver (B) and spleen (C) as described in the Materials and Methods. Each point represents the mean values of the viral titre of three animals, and the standard deviations are shown as error bars.

As shown in Fig. 5, infectious virus was present in the blood, liver and spleen samples from all vaccinated and unvaccinated animals. However, the viral titres of the vaccinated groups were significantly lower than the unvaccinated group (P<0.05). The mean virus titres in the blood from the unvaccinated group was 2x10^6^ PPFU/ml, whereas the titres of the 5, 20, and 40 μg doses were 9x10^3^, 8x10^2^ and 2x10^2^ PPFU/ml, respectively (Fig. 5A). We obtained similar results for the virus titres in the liver and spleen from the vaccinated animals (Fig. 5B and 5C). The infectious titres of the vaccinated animals were approximately 3 to 4 log lower than the unvaccinated animals.

## Discussion

The CCHF virus is one of the most geographically widespread tick-borne viruses. With the increasing number of the cases and expanding new foci of the CCHF endemic area, there is an urgent need to prioritize control strategies on the consideration of disease reduction and its impact on public health. Due to limitations in treatment options and difficulties posed by vector control vaccination remains the most logical method of disease control. Few attempts of vaccine development efforts including inactivated suckling mouse brain viral particles, a DNA-based vaccine and a subunit recombinant vaccine expressing Gn and Gc have been significantly hampered by a lack of animal models and therefore, no protective vaccine has been achieved [17–23]. Immunocompetent mammals, with exception of humans and suckling mice, do not develop signs of disease following CCHFV infection. The CCHF virus infects newborn mice but the disease does not similarity to human CCHF. Recently, IFNAR^−/−^ and STAT1 mice have been established as models of CCHF virus lethal disease resulting from CCHFV infection of IFNAR^−/−^ and STAT-1 mice closely mimics human CCHF [10–12]. Here, we present the results of the study using IFNAR^−/−^ mice, and an exploration of the suitability of this system to test efficacy of the cell culture based vaccine against CCHF.

In the present study, to determine whether IFNAR^−/−^ mice were susceptible to the CCHF virus Turkey-Kelkit06 strain, serial dilutions of the virus were inoculated into IFNAR^−/−^ mice. Infectious virus was detected by PPA at 2 dpi in the blood, liver and spleen (Fig. 1A, 1B and 1C). Consistent with the previous results, the virus titres were independent of the dose of the virus used for the infection [11]. In contrast, when the LD50 of the virus was evaluated in the IFNAR^−/−^ mice, following infection with high doses of the virus, the animals developed a rapid and fatal disease compared to animals receiving lower doses.

After the susceptibility of IFNAR^−/−^ mice to the CCHF virus Turkey-Kelkit06 strain was established, we analyzed the protective efficacy of the cell culture based vaccine against a challenge with 400 LD50 (1,000 PPFU) of the virus. The control mice developed a rapid and fatal disease and were euthanized at 3 days post challenge. In contrast, animals vaccinated with a 5 μg dose of the cell culture based vaccine were partially protected (60% protection), with a significant delay in time to death in the unvaccinated animals (7 days versus 3 days, p<0.01) (Fig. 3D and Table 1). Animals vaccinated with 20 and 40 μg doses of the vaccine had an increased protection of 80% in IFNAR^−/−^ mice and displayed a significantly extended survival time (8 and 9 days, respectively, versus 3 days, p<0.01) (Fig. 4D and Table 1). Recently, Buttigieg et al. developed a poxviral vectored vaccine based on the modified vaccinia virus Ankara (MVA) expressing the CCHF virus glycoproteins [24]. Immunization with the MVA-GP vaccine showed neither morbidity nor mortality to IFNAR^−/−^ mice after lethal challenge with the CCHF IbAr10200 virus. They used 200 TCID50 CCHF virus for the challenge, which was double the minimum lethal dose. In our study, 400 LD50 was used for the challenge, which was 200-fold the minimum lethal dose. Bodur et al. showed that the seroprevalence of CCHF in the endemic region of Turkey was 10%, and approximately 90% of the infections were subclinical, defined as persons who are seropositive although they were not given a diagnosis or had not had severe symptoms compatible with CCHF at any time [25]. There are many factors involved in establishing of the subclinical infections, and the level of viral dose might be one of them. Therefore, these data allowed us to potentially use the high viral dose for the challenge studies.

In the present study, CCHF virus-specific antibodies were observed after the first immunization, while neutralizing antibodies were detected in all animals after the second immunization (Fig. 3B and Table 1). This result showed that at least two immunizations are required for generating neutralizing antibodies. We also determined that IFNAR^−/−^ mice immunized with the cell culture based vaccine induced at high titres of the virus-specific antibodies, indicating that disruption of IFN α/β response has no effect on the humoral arm of the immune system in IFNAR^−/−^ mice. Animals vaccinated with 20 and 40 μg doses of the vaccine exhibited high levels of neutralizing antibodies and displayed 80% protection after the challenge. We concluded that neutralizing antibody responses are essential for the increased of protection of mice vaccinated with the cell culture based vaccine. However, the role of the other the immune responses, such as T-cell responses and antibody-mediated protection should be elucidated.

At 48 hours post challenge, virus titres were detected in the blood, liver and spleen in all of the vaccinated groups. However, the infectious titres obtained from the vaccinated animals were 3 to 4 log lower than the unvaccinated animals (Fig. 4A, 4B and 4C). This result is consistent with patients with lower viral loads who can control viral replication and typically recover 2–3 weeks after disease onset [26–27]. Whether the cell culture based vaccine provides sterilizing immunity in the surviving animals remains unknown.

The results of the present study suggest that immunization with the cell culture based vaccine elicited a significant level of protection against a high dose challenge (1,000 PPFU) with a homologous CCHF virus Turkey-Kelkit06 strain in IFNAR^−/−^ mice. A strong neutralizing antibody response is essential for the increase protection of IFNAR^−/−^ mice immunized with the cell culture based vaccine. In the future, additional experiments will be necessary to evaluate the susceptibility to other CCHF virus strains.
